# Complications of Acute Coronary Syndrome in Young Patients

**Published:** 2017-01

**Authors:** Che’ Muda CHE-MUZAINI, Bachok NORSA’ADAH

**Affiliations:** 1. Dept. of Community Medicine, School of Medical Sciences, Universiti Sains Malaysia, 16150 Kubang Kerian, Kelantan, Malaysia; 2. State Health Dept., Simpang Kuala, Jalan Kuala Kedah, 05400 Alor Setar, Kedah, Malaysia; 3. Unit of Biostatistics and Research Methodology, School of Medical Sciences, Universiti Sains Malaysia, 16150 Kubang Kerian, Kelantan, Malaysia

## Dear Editor-in-Chief

Cardiovascular diseases (CVDs) are the number one cause of mortality throughout the world. More than 20 million people will die mainly from heart disease and stroke by the year 2030 (1). In 2006, “the estimated incidence of ACS was 141 per 100,000 population per year, and the inpatient mortality rate was approximately 7%” (2). In the Euroheart Acute Coronary Syndrome (ACS) survey, 23% of cases were less than 55 years old (3).

ACS refers to a spectrum of clinical presentations ranging from unstable angina (UA) to non ST elevation myocardial infarction (NSTEMI) and to ST elevation myocardial infarction (STEMI). ACS is more prevalent in older patients; thus comparatively few studies have focused on the profiles of ACS in younger patients. The occurrence of ACS in young adults will lead to premature morbidity and mortality in the person’s most productive years of life which also affect his or her family and working life. It may also affect not only physical but psychosocial of the patients.

We conducted a study to identify the characteristics, treatments, complications of ACS in young patients who were admitted to a teaching hospital in Malaysia. Young patients aged less than 45 years old who were diagnosed with ACS based on the symptoms and electrocardiography changes and/or serum biomarkers changes and admitted between 2002 and 2011 were included in this cross sectional study. The cut off age of 45 yr old had been used in most studies to define young patients with ACS (4). The exclusion criteria for this study were patients who were referred or transferred from other hospitals for further management.

The ethical approval was obtained from the Human Research Ethics Committee with reference USMKK/ PPP/ JEPeM (256.4 (1.2). Approval from Director of Hospital was also obtained prior to the study.

A total of 282 patients were included, with a mean age (standard deviation) of 39.2(5.1) years and male to female ratio of 4.5:1. Of the total samples, 46.8% were diagnosed as unstable angina, 29.8% as non ST elevation myocardial infarction and 23.4% as ST elevation myocardial infarction. Most of the patients presented with retrosternal chest pain (72.0%), followed by dyspnea (55.7%), sweating (46.5%) and palpitation (43.3%). The most frequent risk factors of ACS were diabetes mellitus (59.9%), followed by dyslipidaemia (49.3%), current smoking (46.8%), hypertension (43.5%) and heart disease (24.8%). Thirty sixpoint one percent had inpatient complication(s) of ACS with the most common being heart failure (35.4%), followed by arrhythmia (18.4%), cardiogenic shock (14.9%) and pulmonary oedema (9.6%) and death (7.8%). Regarding treatment, only 18.8% had percutaneous coronary intervention, 7.8% had coronary artery bypass graft and the rest received medical treatments. Length of stay ranged from one to 38 days. Only 47.9% had angiogram that showed one vessel disease in 12.4%, two vessels disease in 9.2% and three vessels disease in 9.9%. ST elevation myocardial infarction [adjusted odds ratio (AOR) 2.72; 95% confidence interval (CI): 1.32, 5.64; *P* value = 0.007], hypotension on admission [AOR 9.63; 95% CI: 3.06, 30.29; *P* value < 0.001] and marital status [AOR 3.31; 95% CI: 1.46, 7.49; *P* value = 0.004] were the significant associated factors for ACS complications.

This was a retrospective record review and the disadvantaged of secondary data were missing information, different definition by different attending physicians and assumption of not written as negative behavior. This study was a short term study which may be inadequate to reflect the true burden of premature coronary disease. It should be expanded to other hospitals in order to get more accurate view. With a larger sample size, this study could be divided to each type of ACS because each type might have different associated factors for complications.

In conclusions, ACS in people less than 45 years old in our institution was predominantly males and myocardial infarctions in more than half of cases. About one third of patients had inpatient complication, which is considered relatively high in view to the rapid development of mechanical reperfusion, adjunctive pharmacotherapy, cardiac referral networks and coronary care units. Marital status, ST elevation infarction and hypotensive on admission were the significant risk factors for ACS complication in young patients.
